# Exosomes: key players in cancer and potential therapeutic strategy

**DOI:** 10.1038/s41392-020-00261-0

**Published:** 2020-08-05

**Authors:** Jie Dai, Yangzhou Su, Suye Zhong, Li Cong, Bang Liu, Junjun Yang, Yongguang Tao, Zuping He, Chao Chen, Yiqun Jiang

**Affiliations:** 1 The Key Laboratory of Model Animal and Stem Cell Biology in Hunan Province, Hunan Normal University, Changsha, 410013 Hunan China; 2 School of Medicine, Hunan Normal University, Changsha, 410013 Hunan China; 3 Center for Joint Surgery, Southwest Hospital, Army Medical University (Third Military Medical University), Chongqing, 400038 China; 4 Key Laboratory of Carcinogenesis and Cancer Invasion, Ministry of Education, Department of Pathology, Xiangya Hospital, School of Basic Medicine, Central South University, Changsha, 410078 Hunan China; 5 School of Medicine & Holistic Integrative Medicine, Nanjing University of Chinese Medicine, Nanjing, 210013 Jiangsu China

**Keywords:** Molecular medicine, Cancer microenvironment, Cancer stem cells, Tumour immunology, Tumour biomarkers

## Abstract

Exosomes are extracellular vesicles secreted by most eukaryotic cells and participate in intercellular communication. The components of exosomes, including proteins, DNA, mRNA, microRNA, long noncoding RNA, circular RNA, etc., which play a crucial role in regulating tumor growth, metastasis, and angiogenesis in the process of cancer development, and can be used as a prognostic marker and/or grading basis for tumor patients. Hereby, we mainly summarized as followed: the role of exosome contents in cancer, focusing on proteins and noncoding RNA; the interaction between exosomes and tumor microenvironment; the mechanisms that epithelial-mesenchymal transition, invasion and migration of tumor affected by exosomes; and tumor suppression strategies based on exosomes. Finally, the application potential of exosomes in clinical tumor diagnosis and therapy is prospected, which providing theoretical supports for using exosomes to serve precise tumor treatment in the clinic.

## Introduction

Exosomes, with a size range of 40–160 nanometers in diameter (averaging 100 nanometers), are a subset of extracellular vesicles (EVs) surrounded by a lipid bilayer membrane and secreted by most eukaryotic cells,^1^ Identified as early as in late 1980s, exosomes were originally and simply considered as cellular waste products.^2^ However, with the development of research methodologies and techniques, people now have realized that exosomes represent a novel mode of intercellular communication and contribute to a wide range of biological processes in health and disease including cancer.^3^ The biological function of exosome relies on its bioactive cargos, such as lipids, metabolites, proteins and nucleic acids,^4–7^ which can be delivered to the target cells. Growing evidence suggests that tumor-derived exosomes (TEXs) play critical roles in cancer. Exosomes and their cargos may serve as cancer prognostic marker, therapeutic targets or even as anticancer drug‐carrier.^8^ In this review, we endeavor to summarize the bioactive exosomal contents focusing on proteins and noncoding RNAs, clarify the crosstalk of exosome with tumor microenvironment (TME), elucidate the underlying mechanism of affected epithelial-mesenchymal transition (EMT), invasion and migration affected by exosomes, and discuss the future tumor suppression strategies based on exosomes.

## Exosomes biogenesis and isolation

### Biogenesis

**Fig. 1:**
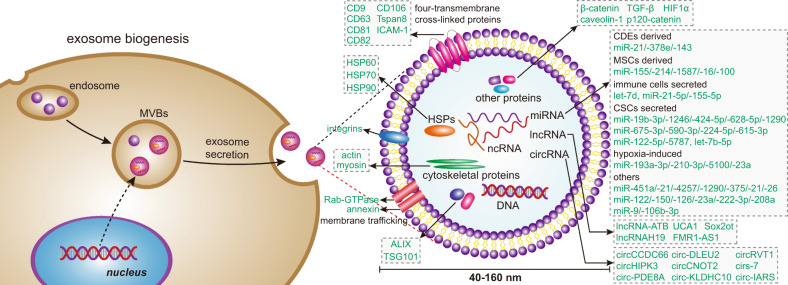
Exosome biogenesis and its contents. Exosomes originated from MVBs, which contain many kinds of proteins, such as membrane transporters, HSPs and so on. In addition, it also contains a lot of noncoding RNA, including miRNA, lncRNA, and circRNA. These contents play an important role in the development of tumor. MVBs multi-vesicular bodies, CDEs cancer-associated fibroblast-derived exosomes, MSCs mesenchymal stromal cells, CSCs cancer stem cells

Exosomes are originated from the endocytic pathway.^9^ A typical process of exosomes formation comprises the following steps (Fig. 1): (i) the cytoplasmic membrane invaginates to form an early secretory endosome; (ii) followed with the payload sprouts inward to form intraluminal vesicles (ILVs) contained within the endosome, which termed a multi-vesicular bodies (MVBs) biogenesis; (iii) then, the late endosomes maturation by acidification; (iv) and eventually extracellular release of ILVs as exosomes by fusion with the plasma membrane.^10^ It is known that the endosomal sorting complex required for transports (ESCRTs) mechanism plays an important role in the process of MVBs and ILVs biogenesis. The components of ESCRTs (containing ~20 proteins) comprises four complexes, termed ESCRT-0, -I, -II, and -III, with associated proteins, including ALIX (Apoptosis-linked gene 2-interacting protein X, encoded by PDCD6IP), VTA1(Vesicle Trafficking 1), VPS4 (Vacuolar protein sorting-associated protein 4), and TSG101 (Tumor susceptibility gene 101 protein).^11,12^ During the process of MVBs biogenesis, the ESCRT-0 complex is recruited by ubiquitinated cargo, the major pathway-specific signal in MVBs biogenesis, to the endosomal membrane; the ESCRT-I and -II components make membrane deformation into buds and isolate the payload; and the ESCRT-III complex separates the vesicles from the cytoplasmic membrane, subsequently.^13,14^ In addition, other ESCRTs-independent mechanisms have been found to affect the formation of exosomes, such as neutral sphingomyelinase 2-dependent pathway, heterogeneous nuclear ribonucleoprotein-dependent pathway, miRNA post-transcriptional 3′end modification and RNA induced silencing complex related pathway.^15^

### Isolation

Exosomes are ubiquitous in healthy or pathological organisms. The presence of exosomes in urine, serum, plasma, lymph, or cerebrospinal fluid from the healthy person or cancer patient was confirmed.^16,17^ Some classical approaches for exosomes isolation from body fluids or cell culture supernatants have been reported, like ultracentrifugation, ultrafiltration and immunoaffinity capture-based techniques.^18^ To further increase the purity, and efficiency of exosome isolation, some emerging and interdisciplinary technologies have been applied for exosomal preparations. The microfluidics-based platforms for exosome isolation is one of them, utilizing the physical and biochemical properties of exosomes at microscales. In addition to canonical approaches, like size, density and immunoaffinity based isolation, novel sorting mechanisms such as acoustic^19^ and electromagnetic manipulations^20^ have been employed. With these isolation platforms, the acquisition of exosomes will be much easier and the sample, reagent, and time consumption in isolation process will be significantly reduced.

## Contents of exosomes

Exosomes comprise a variety of substances, containing 9769 proteins, 3408 mRNAs, 2838 miRNAs, and 1116 lipids, according to the latest update lists of the exosome database (http://www.exocarta.org).^21^ Its components can act as autocrine and/or paracrine factors, including specific lipids, proteins, DNA, mRNA, and noncoding RNAs (Fig. 1). These contents of exosomes can be used as prognostic markers and/or graded basis for cancer progression. It also regulates tumor growth, metastasis, angiogenesis, and mediates drug resistance in tumor cells.^16^ Some research process of exosomal components like proteins and noncoding RNAs will be described as follows:

### Exosomal proteins

Exosomes, as a member of the EVs, participate in physiological and pathological processes by delivering a vast array of signaling molecules, such as, mRNAs, miRNAs, nucleic acids, lipids, and proteins. Exosomal proteins have a specific characteristic called ubiquitination which allows proteins to be recognized by ESCRT-0, whereas deubiquitination is a crucial step for sorting them into ILVs. But it is still remain controversial that whether ubiquitination is mandatory for driving proteins into exosomes.^22^ Exosomal proteins include: (i) Membrane transport and fusion related proteins like annexin, Rab-GTPase (Ras-related protein GTPase Rab), and heat shock proteins (HSPs) including Hsp60, Hsp70, and Hsp90; (ii) Tetraspanins (also termed four-transmembrane cross-linked proteins), including CD9, CD63, CD81, CD82, CD106, Tspan8, ICAM (intercellular adhesion molecule)-1; (iii) MVBs related proteins, for instance, ALIX and TSG101 (the stereotypical biomarker for exosomes characterization); (iv) other proteins, like Integrins (cell adhesion-related proteins), actin and myosin (participating in cytoskeletal construction).^23–25^ The above-mentioned proteins play a crucial role in exosomes. Tetraspanins have been indicated to facilitate the entry of specific cargos into exosomes, CD9 mediates the metalloproteinase CD10 loading into exosome.^26^ As members of MVBs related proteins, ALIX and TSG101 are known components of ESCAT machinery and classify the cargo proteins of ILVs by recognizing the ubiquitinylated proteins and then arrange them on the plasma membrane as the components of exosomes.^27^ HSPs facilitate protein folding and balance of proteostasis and proteolysis acting as the molecular chaperones and play anti-apoptotic roles in tumors.^28^ Among the HSPs, Hsp90 is the major intercellular chaperones that ensures proper proteins folding and function by interacting with a variety of intracellular proteins. The high-expression of Hsp90 in various cancer cells because of the fact that tumor cells are constantly in a state of stress like hypoxia, acidosis, metabolic and nutrient deficiency. And Hsp90 plays a crucial role in promoting tumor growth and metastasis of breast cancer, pancreatic cancer (PC), leukemia, and closely associated with poor prognosis of tumors.^29^ Additionally, for exosome-related functions, a recently research showed that membrane deformability of Hsp90 mediates fusion of MVBs and plasma-membrane.^30^ The exosomes lack of extracellular Hsp90α, a key subtype of Hsp90, will lose the capacity to carry out the important intercellular communication from tumor cells to stromal cells, which promoting cellular motility.^31^

### Exosomal noncoding RNAs

#### miRNA

microRNAs (miRNAs), as an important members of small noncoding RNAs with length between 20 and 22 nucleotides, mediate post-transcriptional gene silencing by combining with the 3′-untranslated region or open reading frames of the target mRNA, and have been extensively studied in various physiological and pathological processes.^32,33^ During the developmental processes of cancer, miRNAs in exosomes can serve as the potential biomarkers for cancer prognosis and/or grading basis. Exosomal miR-451a, miR-21, and miR-4257 were discovered abnormally high expressed in non-small cell lung cancer patients and strongly associated with tumor progression, recurrence, and poor prognosis.^34,35^ Analysis of plasma EV samples from prostate cancer patients revealed that let-7a-5p from plasma EVs was significantly lower in prostate cancer patients with high GS (Gleason score) compared to those with low GS.^36^ Combined analysis of exosomal miR-1290 and miR-375 has been reported to be able to predict overall survival of castrate-resistant prostate cancer patients. Over the same follow-up period of 20 months, patients with a high level in both miRNAs had a general mortality of 80%, while patients with a normal concentration in both only had a mortality rate of 10%.^37^ Sun et al. isolated and analyzed exosomes secreted by cancer stem cells (CSCs) and corresponding parental cells, and found six miRNAs (miR-1246, miR-424-5p, miR-628-5p, miR-1290, miR-675-3p, and miR-590-3p) were upregulated and five miRNAs (miR-224-5p, let-7b-5p, miR-615-3p, miR-122-5p, and miR-5787) were down-regulated, which could be expected to be biomarkers for potentially predicting the patient with high risk for developing gastric cancer and diagnosing gastric cancer at an early stage.^38–40^

Furthermore, miRNAs can be detected in exosomes isolated from body fluids (such as saliva, blood/serum), indicating the potential advantages of using exosomal miRNAs as non-invasive novel biomarkers.^41^ Multiple miRNAs including miR-21, miR-26, miR-122, and miR-150 had been identified as blood-based biomarkers for the non-invasive diagnosis of cholangiocarcinoma.^42^ For exosomal miRNAs in lung cancer research, Hu et al. summarized the exosomal miRNAs in lung cancer as the diagnostic, predictive and prognostic biomarkers.^43^ Exosomes isolated from the plasma of 45 non-small cell lung cancer patients and 31 controls were analyzed and indicated that exosomal miR-126 was upregulated and could be the diagnostic biomarker for non-small cell lung cancer.^44^ Elevated levels of circulating exosomal miR-23a are found in the sera of lung cancer patients, and miR-23a levels are positively correlated with proangiogenic activities, which could also be used as a biomarker for the diagnosis of lung cancer.^45^ Apart from the diagnostic biomarkers, exosomal miR-222-3p could be employed as the predictive biomarker for gemcitabine (GEM) sensitivity^46^ and exosomal miR-208a as the predictive biomarker for radiation responses.^47^

miRNAs in exosomes could also promote tumor progression in a variety of ways. miR-9 in exosomes derived from triple-negative breast cancer cells could stimulate the migration of tumor cells by down-regulating E-cadherin of normal fibroblasts (NFs), and promoted the transformation of cells from NFs into cancer-associated fibroblasts (CAFs).^48^ TEXs could promote tumor development by transferring miRNA-mediated Ca^2+^ receptor instability.^49^ In addition, exosomal miRNAs from mesenchymal stromal cells (MSCs) or fibroblasts had been shown to be delivered to tumor cells directly to promote cancer progression and induce drug resistance in multiple myeloma (MM), colorectal and gastric cancer cells.^50–52^

#### lncRNA

Long noncoding RNA (lncRNA) is transcript longer than 200 nucleotides and lack important open reading frames. It plays an important roles in many life activities, such as dose compensation, cell cycle regulation, epigenetic regulation, and cell differentiation regulation.^53^ It is an emerging regulatory RNA that can be selectively packaged into exosomes and acts as a messenger in intercellular communication to regulate tumor growth, metastasis and angiogenesis, and reshapes the TME.^54,55^ For example, lncRNAs-ATB was a novel cancer-related lncRNA that was abnormally expressed in hepatocellular carcinoma (HCC), colorectal cancer (CRC), gastric cancer, and lung cancer, and promoting tumorigenesis and development mainly by competitively binding miRNA (miR-200 family) to induce EMT.^56^ Conigliaro et al. found that exosomes secreted by CD90 cells and CSCs could induce angiogenesis in HUVECs (human umbilical vein endothelial cells). The exosomes could subsequently be ingested by endothelial cells, and delivering lncRNA H19 to the corresponding target cells by adhesion to CD90 cells and HUVECs, and stimulating angiogenesis by the synthesis and release of vascular endothelial growth factors.^57^ lncRNA also plays an important role in tumor resistance.^58^ UCA1 (a lncRNA) activated the Wnt signaling pathway and promoted cisplatin resistance in bladder cancer cells by increasing the expression of Wnt6. Thus, UCA1/Wnt6 pathway was a potential target for bladder cancer resistance.^59^

#### circRNA

Another type of RNA in exosomes, circular RNA (circRNA), is a new family of noncoding endogenous RNA found in all eukaryotic cells. It has a tissue-specific and cell-specific expression pattern covalently blocked endogenous biomolecules whose biogenesis is regulated by specific cis-acting elements and trans-acting factors.^60^ circRNA is produced by a specific mechanism called "reverse splicing" for alternative splicing. It forms a special class of noncoding RNAs without the cyclic structure of 5′-3′ polarity and polyadenylation tails, with intrinsic resistance to nucleic acid-degrading enzymes that target 5′ and 3′ ends.^61,62^ circRNAs have been shown to competitively bind to miRNA as endogenous RNA, inhibiting the process of miRNA targeting mRNA.^63^ Many circRNAs performed important biological functions by acting as miRNA competitively inhibitors to regulate protein function or their own translation, such as circCCDC66, circHIPK3, circPVT1, and cirs-7 in cancer.^57^ circRNAs exhibits multiple functions by acting as regulators of gene and miRNA expression, and can play a role in several cancer biological processes, including tumor cell proliferation, invasion, metastasis, and progression. Circ-IARS expression was upregulated in the plasma exosomes of patients with in-situ and metastatic PC. The overexpression of circ-IARS significantly down-regulated the levels of miR-122 and ZO-1, upregulated the levels of RhoA and RhoA-GTP, increased the expression and adhesion of F-actin, enhanced the permeability of endothelium, and promoted tumor invasion and metastasis.^64^ Moreover, The upregulation of circ-DLEU2 promoted the expression of PRKACB by inhibiting the expression of miR-496, which promoted the proliferation of leukemia cells in vitro, blocked cell apoptosis, and promoted the formation of acute myeloid leukemia tumors in vivo.^65^

In summary, since the expression levels of circRNAs are generally associated with clinicopathologic characteristics, these RNAs may serve as the biomarkers with diagnostic, prognostic, and predictive properties. And the high stability of circRNAs may allow non-invasive detection of them in body fluids.^60^ For instance, circCNOT2 in primary breast cancer had been shown to be detectable in the plasma of breast cancer patients, suggesting that circCNOT2 could work as a useful biomarker for selecting suitable treatment strategy or for minimally-invasive progression monitoring.^66^ For the detection of circ-KLDHC10 in the exosomes of CRC patients, the expression level of circ-KLDHC10 in serum exosomes was compared to distinguish colon cancer patients from healthy controls.^67^

## Relationship between exosomes and tumor microenvironment

**Fig. 2:**
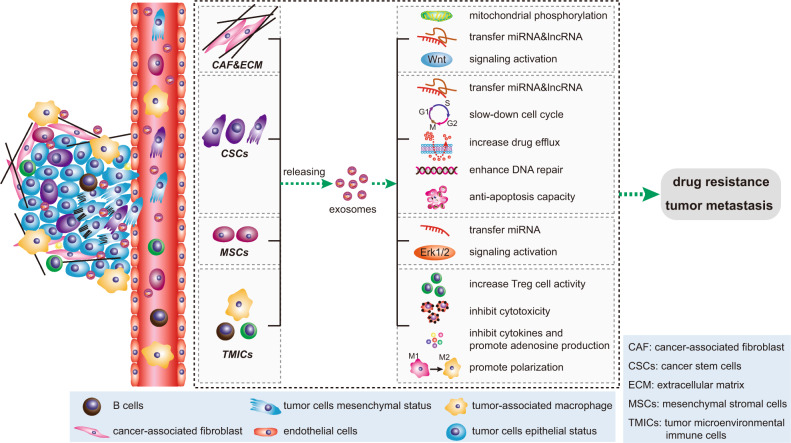
Signal transduction pathway of exosomes in tumor microenvironment. The most important cells in tumor microenvironment mainly include CAFs, CSCs, MSCs, TMICs, etc. These four types of cells use exosomes to promote EMT, tumor metastasis and drug resistance through a variety of mechanisms. CAFs cancer-associated fibroblasts, CSCs cancer stem cells, MSCs mesenchymal stromal cells, TMICs tumor microenvironmental immune cells

TME consists of extracellular matrix, stromal cells (including fibroblasts, MSCs, pericytes, occasional adipocytes, blood, and lymphatic network) and immunity cells (including T and B lymphocytes, natural killer cells, and tumor-associated macrophages).^68^ TME plays an indispensable role in tumor biology and is involved in tumorigenesis, progression and response to treatment. Exosomes are an important part of TME.^69^ They act as effective signaling molecules between cancer cells and the surrounding cells that make up TME.^70^ In the following, we will focus on the important cells of the TME and their important links with exosomes (Fig. 2).

### Exosome and cancer-associated fibroblasts

CAFs are the main cellular components of TME in most solid cancers.^71^ CAF-derived exosomes (CDEs) are one of the key factors of oncogenic transformation.^72^ CDEs promotes the growth of cancer cells by inhibiting mitochondrial oxidative phosphorylation, thereby increasing glycolysis and glutamine dependent reduction carboxylation in cancer cells.^71^ In breast cancer patients, the expression levels of miR-21, miR-378e, and miR-143 in CDEs were higher than those in NFs. Breast cancer cells transfected with these three miRNAs could promote the stemness and EMT of these cells.^73^ CDEs not only enhance cancer growth, but also promote drug resistance and tumor metastasis.^74^ For example, CDEs could promote neoplastic angiogenesis and tumor development in CRC, and could also induce the dedifferentiation of cancer cells through the Wnt pathway, thereby promoting the chemical resistance of CRC.^75,76^ Studies have found that CAFs were inherently resistant to gemcitabine (GEM, chemotherapy standard for pancreatic ductal adenocarcinoma). CAF exposure to GEM significantly increased the release of exosomes, thereby increasing cell proliferation and survival of recipient epithelial cancer cells.^77^ In addition, CAFs produce exosomes with high level of TGF-β1,^78^ which is capable to induce metastatic activity of cancer cells by regulating the expression of lncRNA.^79^ Yu. et al. reported that TGF-β1 was top one highest level of cytokine secreted by CAFs, which was essential for CAFs-induced EMT and metastasis in breast cancer cells. CAF-derived conditional medium significantly increased the HOTAIR expression to promote EMT of breast cancer cells. However, this could be abrogated by treatment with TGF-β1 inhibitor SB451332 or Pirfenidone. Moreover, depletion of HOTAIR inhibited CAFs-induced tumor growth and lung metastasis in MDA-MB-231 orthotopic animal model.^80^ In HCC, overexpression of lncRNA-ATB further promoted metastasis of hepatoma cells by inducing EMT and invasion.^81^

### Exosomes and cancer stem cells

CSCs, also known as cancer-initiating cells, are a small subset of heterogeneous cells present in tumor tissue. CSCs have unlimited self-renewal and diversification potential and play an important role in tumor initiation, recurrence, metastasis, and therapeutic resistance.^82^ As information carriers, exosomes are involved in the transformation between non-CSC and CSC as well as the maintenance of CSC homeostasis and its mechanism.^23^ lncRNA FMR1-AS1 in exosomes could maintain the dynamic interconversion state of CSCs by activating TLR7-NFκB signaling.^83^ The exosomes derived from CSC transferred miR-19b-3p to clear cell renal cell carcinoma cells, and then initiated EMT to promote tumor metastasis.^84^ In contrast, p120-catenin in exosomes secreted by HCC (hepatocellular carcinoma) cells inhibited the proliferation, metastasis, and expansion of HCC CSCs.^85^ Exosomes are also important participants in the resistance of CSCs, which involving multiple mechanisms, including enhanced DNA repair efficiency and anti-apoptotic capacity, slow cell cycle progression, drug efflux, and expression of detoxifying enzymes.^86^ For example, in PC, BxR-CSC (GEM-resistant BxPC-3 cells)-derived exosomes induced GEM resistance, inhibited GEM-induced cell cycle arrest, and antagonized GEM-induced apoptosis. It also promoted tube formation and cell migration of BxS (GEM-sensitive BxPC-3 cells) and PANC-1 cells.^87^ In breast cancer, RAB27B promoted exosomes transfer from stromal cells to breast cancer cell with the transfer of exosomal 5′-triphosphates which activated the RIG-I (retinoic acid-induced gene 1 enzyme) signal in target cells, thereby activating IRDS (Interferon-Related DNA Damage Resistance Signature) genes, in parallel activation of the NOTCH3 pathway to regulate the expansion of therapy-resistant tumor-initiating cells known as CSCs.^88^

### Exosome and mesenchymal stem cells

MSCs are considered to be one of the most promising seed cells in tissue engineering because of their easy availability and pluripotency characteristics of adipocytes, osteoblasts, cardiomyocytes, and neurons.^89^ Exosomes derived from MSCs in the TME facilitate the conversion of non-CSC to CSC. Exosomes released by MSCs confer colorectal stem cell phenotypes by activating the Wnt signaling pathway, including tumor spheroid formation in vitro and tumorigenicity in vivo, increasing the percentage of CSCs, and activating ERK1/2 (extracellular signals to regulate kinase 1/2), thus, promoted tumor growth and progression.^90^ In tumors, MSC-derived exosomes regulate tumor markers and help tumor progression by delivering special miRNA to neighboring cells. For example, bone marrow-derived MSCs released exosomes containing miR-214 through CaMKII silencing to inhibit oxidative stress injury in CSC, thus helping tumor progression.^91^ Exosomes from tumor-associated MSCs contained high levels of miR-155, which, after being taken up by atypical teratoid/rhabdoid tumor cells, could cause tumor suppressor genes SMARCA4 (direct target genes of miR-155) inhibition, and enhanced atypical teratoid/rhabdoid tumor migration ability.^92^ In addition, glioma-associated human MSCs, a potential new matrix component in glioma, could drive the invasibility of glioma stem cells (GSCs) and have been identified as a new therapeutic target in glioma.^93^ miR-1587 in glioma-associated human MSCs derived exosomes promoted proliferation and clonal formation in GSCs by down-regulating the tumor-suppressive nuclear receptor corepressor NCOR1 in GSCs.^94^ However, it is interesting that MSC exosomes also contain anti-angiogenic miRNAs, such as miR-16 and miR-100, which inhibit angiogenesis by targeting vascular endothelial growth factor in breast cancer cells in the TME.^95–97^ In addition, MSC-derived exosomes also play an important role in tissue repair and inflammatory response.^98^

### Exosome and tumor microenvironmental immune cells

The tumor microenvironmental immune cells mainly include myeloid cells (tumor-associated macrophages, dendritic cells (DCs), myeloid-derived suppressor cells, etc.) and lymphocytes (T and B cells).^99^ Tumor cell-derived exosomes are a good source of cellular components that stimulate the immune response, such as alarmins (mRNA, transmembrane proteins including CD9, CD63, and CD81, HSPs, major histocompatibility complex I molecules) and tumor-associated antigens.^100^ The antigen-presenting and immune-stimulating properties of TEXs enable them to trigger antitumor responses and contribute to the recruitment and reconstruction of tumor microenvironmental components.^101^ For example, TEXs derived from oral squamous cell carcinoma and histiocytic lymphoma could induce immunosuppression through the CD95 (Fas) receptor and FasL^+^ exosomes signaling on activated CD8^+^ T cells.^102,103^ PC-derived exosomes switched the differentiation of macrophages to the M2 phenotype, thereby promoting the immunosuppression and metastasis of HIF-1α or HIF-2α.^104,105^ Exosomes derived from Lewis lung cancer or 4T1 breast cancer cells prevented the differentiation of myeloid precursor cells to CD11c^+^ DCs and induced apoptosis.^106^ Under hypoxic conditions, tumor cells released exosomes with TGF-β, which could also promote Treg cell activity and inhibited NK cell cytotoxicity to form an immunosuppressive environment.^107,108^ Tumor microenvironmental immune cells also produce exosomes. Treg cells prevented the maturation of antigen-presenting cell by expressing CTLA-4, or produced inhibitory cytokines and adenosine anti-tumor immunity to participate in tumor development and development.^109^ Treg-derived exosomes had also been implicated in immunosuppressive functions.^110,111^ Treg cell exosomes miRNA (Let-7d) strongly inhibited Th1 cell activity by inhibiting COX-2-mediated IFN-γ production.^112^ Macrophages are the major immune cells that regulate inflammation in the TME, including two broad phenotypes, M1 and M2. M1 macrophages can kill viruses and bacteria by secreting IL-12 and IL-23 inflammatory factors. M2 macrophages can promote blood vessel formation and tumor growth and metastasis.^113^ In CRC, exosomes secreted by M2 macrophages highly expressed miR-21-5p and miR-155-5p to regulate the migration and invasion of CRC cells.^114^

### Exosomes affect EMT, invasion and migration

Exosome signaling can induce the production of CSCs through EMT in the TME and generate favorable treatment resistance conditions.^115^ EMT refers to the biological process by which epithelial cells are transformed into cells with mesenchymal phenotypes through specific programs. EMT regulation requires a complex transcription mechanism, mainly composed of developmental transcription factors, which can be divided into three categories: SNAIL family of zinc-finger transcription factor SNAIL/SLUG, ZEB1/ZEB2 ZEB (zinc-finger e-box binding homogenic box) family, and TWIST1/TWIST2 family.^116^ In addition to transcriptional factor, EMT regulation also involves multiple noncoding RNA inducers, including miRNAs and lncRNAs.^117–119^ TEXs are likely to carry these inducer molecules to facilitate the EMT process.

In examining the biological function of exosomes from highly metastatic lung cancer cells and human advanced lung cancer, researchers found that those exosomes can increase Vimentin expression, promote EMT, invasion and migration of target cells.^120^ Exosomal miR-106b-3p from CRC cells was found abundant in metastatic CRC patients’ serum and correlated with poor prognosis. Further research demonstrated that exosomal miR-106b-3p can increase lung metastasis of CRC cells via Vimentin, N-cadherin upregulation and E-cadherin down-regulation by targeting DLC-1 (Deleted in Liver Cancer-1) gene.^121^ In pancreatic ductal carcinoma(PDAC), lncRNA-Sox2ot was identified from highly invasive PDAC cells, and plasma exosomal Sox2ot expression was closely associated with TNM stage and overall survival rate of PDAC patients. Exosomal Sox2ot works as ceRNA who can competitively bind with the miR-200 family to regulate SOX2 expression, which increased EMT and stem cell-like properties, resulted in promotion of invasion and metastasis of PDAC.^122^ It was reported that PDAC cell-derived exosomal circ-PDE8A acts as a ceRNA for miR-338 to regulate MACC1 and stimulates invasive growth via the MACC/MET/ERK or AKT pathways. Moreover, exosomal circ-PDE8A expression in plasm of PDAC patients was related with progression and prognosis, thus suggest it may play an important role in tumor invasion, could be a potential marker for PDAC diagnosis or progression.^123^

TEXs can carry proteins including TGF-β, caveolin-1, HIF1α, and β-catenin, which enhanced invasiveness and the ability of target cells to migrate, and helped matrix remodeling and niche formation.^124^ Exosomes derived from gastric cancer cells could induce the differentiation of human umbilical cord-derived MSCs to CAFs by transferring TGF-β and activating TGF-β/Smad pathway, which assisting tumor niche formation.^125,126^ It has been demonstrated that the presence of caveolin-1 in prostate cancer-derived exosomes could potently induce CSC phenotype and EMT process via NF-κB signaling.^127^ Exosomes containing high expression of caveolin-1 could promote migration and invasion of tumor cells lacking caveolin-1.^128^ Aga et al. detected endogenous HIF1α in nasopharyngeal carcinoma-derived exosomes and found that exosomes treatment of LMP1 (latent membrane protein 1), a principal oncoprotein of EBV (Epstein-Barr virus), obviously enhanced the levels of HIF1α in exosomes and increased migration and invasiveness of EBV-negative nasopharyngeal carcinoma cells.^129^ Additionally, HCC cells released exosomes contained β-catenin, and Vps4A, a classical tumor suppressor, could downregulate exosome release of β-catenin and inhibit EMT and motility of HCC cells via inhibiting β-catenin signaling.^130^ The CRC cells secreted exosomes could transfer mutated β-catenin to recipient cells activating Wnt signaling and enhance tumor burden in xenograft mice models.^131^

TGF-β and WNT/β-catenin signaling pathways are key regulators in EMT.^132–134^ Exosomes derived from HCC cells could mediate EMT through activating TGF-β/Smad signaling pathway, inducing a decrease in E-cadherin expression, but an increase in Vimentin, which resulted in promoted migration and invasion of target cells.^135^ The TGF-β1/Smads pathway was an important mechanism for bone marrow-derived MSCs to induce EMT in breast cancer cells.^136^ The Wnt/β-catenin signaling pathway and Wnt activity are associated with ovarian cancer classification, EMT, chemotherapy resistance, and poor prognosis. In addition, the ERK protein subfamily made a huge contribution to EMT and was overexpressed and overactive in various types of cancers.^137^ Pancreatic stellate cells, typically activated in pancreatic ductal adenocarcinoma, -derived exosomes increased the expression of ERK1/2 (extracellular signals to regulate kinase 1/2) in PDAC cells, which leading to promote cell migration, EMT, and enhance matrix metalloproteinase‑2/9 activity.^138^ Highly metastatic HCC MHCC97H cells-derived exosomes could communicate with low metastatic HCC cells and subsequently enhanced its migration, invasion, chemotaxis, and EMT by ERK signaling.^139^

It is very interesting that hypoxia is a very important factor involving in exosomes regulated EMT. Exosomes derived from hypoxic bone marrow-derived MSCs mediated transfer of miRNAs, including miR-193a-3p, miR-210-3p and miR-5100, from bone marrow-derived MSCs to lung cancer cells, increased the expression of total and phosphorylated STAT3, thus promoted invasion of lung cancer cells by activating STAT3 signaling-induced EMT.^140^ Hypoxia-induced CRC-derived exosomes, containing miR-23a, which could lead to the down-regulation of PDH1/2 when applied to CRC cells, thus eventually upregulated HIF-1α, an independent activator of EMT.^141^

## Exosome-based tumor suppression strategies

### Exosomes as drug carriers-targeted inhibition of tumor cells

**Fig. 3:**
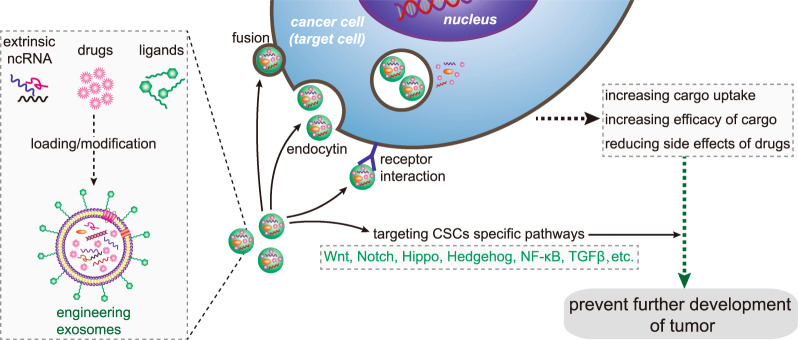
Precisely targeting tumor with engineering exosomes as delivery carrier. Exosomes are carriers with natural delivery ability, which have the characteristics of precisely targeting and high bioavailability. After being loaded into exosomes, anticancer drugs and/or extrinsic ncRNA can directly target cancer cells or CSCs specific pathways, and prevent the further development of tumors. Additionally, the surfaces of exosomes can also be modified with the ligands corresponding to receptors overexpression on cancer cell surfaces, which improving cellular uptake efficiency of exosomes by cancer cells. ncRNA noncoding RNA, CSCs cancer stem cells

In order to improve the effectiveness of cancer treatment, we urgently need to accurately deliver drugs to tumor cells. Clinically, nanotechnology-based drug delivery systems are one of the most promising tools to achieve this goal. Using its natural delivery capabilities, exosomes have been successfully used as drug and functional RNA delivery vectors in cancer treatment.^142^ Exosomes can be absorbed by cells and can stably transfer drugs, such as therapeutic miRNAs and proteins^143^ (Fig. 3). Compared with liposome nanomaterials, metal nanomaterials, and polymer nanomaterials, exosomes as carriers can overcome the shortcomings of poor bioavailability and reduce non-targeted cytotoxicity and immunogenicity.^144,145^ And exosomes contain transmembrane and membrane anchoring proteins, which enhance endocytosis and thus promote the transfer of their contents.^146^ For instance, Kim et al. found that paclitaxel-loaded macrophage-derived exosomes significantly increased cell uptake in 3LL-M227 mouse Lewis lung cancer cell line, compared to paclitaxel-loaded liposomes.^147,148^ Compared with free drugs in animal tumor models, exosome-mediated chemotherapy delivery can enhance drug effects. For example, the antimitotic chemotherapy drug paclitaxel could be loaded into exosomes by sonication. And these loaded exosomes were 50 times more cytotoxic to drug-resistant cancer cells in vitro than free paclitaxel.^148^ Additionally, compared with free drugs, exosome-based delivery platform can greatly reduce side effects. Studies have shown that exosomes coated with different chemotherapeutic drugs were transported to the tumor tissues of mice and could inhibit tumor growth, but no equivalent side effects of free drug have been observed.^149^ The use of engineered exosomes containing miR-21 sponge constructs had the potential to downregulate the expression of miR-21 in glioma cell lines U87-MG and C6, thereby up-regulating the target genes PDCD4 and RECK of miR-21 and preventing their malignant behavior.^150^ Recent studies have shown that exosome surface modification is performed using oligonucleotide binding methods. Such cargo may not only potentially alter cell function, but also alter cell-to-cell transport.^151^ Triple-negative breast cancer is one subtype of breast cancer and with the most metastatic and recurrent characteristic. Li et al. modified the surface of the exosomes with a peptide targeting mesenchymal-epithelial transition factor gene (c-Met), a tyrosine kinase receptor for hepatocyte growth factor or scatter factor, which is overexpressed on triple-negative breast cancer cell surfaces.^152^ These engineered exosomes showed significantly improved cellular uptake efficiency and antitumor efficacy of doxorubicin.^153^

Exosomes can also affect CSCs by targeting CSC-specific signaling pathways, such as Wnt, Notch, Hippo, Hedgehog, NF-κB, and TGF-β pathways. These pathways are of great significance for maintaining a series of biological functions, like self-renewal, differentiation, and tumorigenesis of CSCs. Selective targeting of CSCs via the above pathway using exosome loading inhibitors (miRNA or siRNA) is considered to be achievable.^86^ Lymphoma side population cells outputted Wnt signaling pathway agonist Wnt3a to neighboring cells through exosomes, thereby regulating the number balance of tumor cell population.^154^ Existing results have shown that exosomal Wnt from fibroblasts could induce dedifferentiation of cancer cells to promote chemotherapy resistance in CRC, and suggesting that interference with exosomal Wnt signaling could help improve chemosensitivity and treatment window.^75^

### Anti-tumor vaccine using exocrine system

TEXs have a dual effect on the immune system, i.e., immunosuppressive or immunostimulatory effects. Numerous research have shown that TEXs can interfere with the maturation of DCs, weaken the activation of NK cells, induce suppressor cells of myeloid origin, and transform macrophages into tumor-promoting phenotype.^155–157^ The activated CD8^+^ effector T cells in the circulation system of cancer patients were induced apoptosis by TEXs, which was one of many immunoinhibitory mechanisms of TEXs and suppressed patient’ general immune system.^72^ Exosome as a carrier for delivery products can initiate antitumor immune responses with significant therapeutic effects on tumor progression.^158^ In mouse model with melanoma, mice were treated with α-galactosylceramide/ovalbumin-loaded exosomes, which induced an early T cell response and eventually slowed tumor growth compared to the control group.^143^ Abundant alpha fetoprotein in exosomes produced by in vitro cultured HCC could stimulate the antigen-presenting function of DCs, stimulate the proliferation of CD8^+^ T cells, regulate the secretion of inflammatory cytokines (reducing IL-10 and TGF-β secretion and increasing secretion of IFN-γ and IL-2), and enhance immune-induced apoptosis.^159–161^ According to the study of Xie et al., a vaccine developed by exosomes was effective in antitumor immunity. In their study, exosomes from MM (multiple myeloma) cells were used to stimulate anti-tumor immune responses and generate prophylactic immunity in MM cell lines.^162,163^ TEXs recovered and enriched from patient sera may well provide an optimized, individual-specific source of antigen for DCs vaccination.^164^ How to make full use of the advantages of TEXs, and bypass their disadvantages to regulate tumor immunity needs further research, which has great potential in the application of cancer targeted therapy.^165^

## Conclusions and perspectives

Cancer-related exosomes are produced by cancer cells, CSCs or tumor microenvironment associated cells. They contain numerous categories of contents such as proteins, DNA, mRNA, miRNA, lncRNA, and circRNA. Some of them act as biomarkers, we can take this advantage for cancer early detection, early diagnosis, prognosis prediction, and therapeutic efficacy evaluation. Some of the contents act as mediators of signal transduction between cancer cells with cancer cells, or cancer cells with tumor microenvironment associated cells, and contribute to tumor development, invasion, metastasis, and drug resistance. Based on this, we can develop new strategies relying on engineered exosomes carrying with tumor-suppressing proteins, nucleic acid components or targeted drugs function as precision medicine.

Although there have been many studies trying to clarify the molecular mechanism of how exosomes are produced, endocytosis and play biological roles in tumor progression, more efforts are still needed to further elucidate these problems to make the diagnostic and therapeutic potential of exosomes a clinical reality. We believe that with the continuous research work, in the near future, we may make full use of the advantages of exosomes as natural carriers and bypass the shortcomings. Great progress will be made in cancer treatment strategies based on exosomes, for the great goodness of vast cancer patients.
